# Alpelisib Therapy in 2 Patients With Congenital Hyperinsulinism

**DOI:** 10.1210/jcemcr/luaf099

**Published:** 2025-06-09

**Authors:** Khalid Alburshad, Rasha Amin, Hajar Dauleh, Marwa Ibrahim, Khalid Hussain

**Affiliations:** Endocrinology Department, Sidra Medicine, PO BOX 26999, Doha, Qatar; Endocrinology Department, Sidra Medicine, PO BOX 26999, Doha, Qatar; Endocrinology Department, Sidra Medicine, PO BOX 26999, Doha, Qatar; Endocrinology Department, Sidra Medicine, PO BOX 26999, Doha, Qatar; Endocrinology Department, Sidra Medicine, PO BOX 26999, Doha, Qatar

**Keywords:** congenital hyperinsulinism, alpelisib, hypoglycemia, Usher syndrome, *KCNJ11* pathological variant

## Abstract

Congenital hyperinsulinism (CHI) is a disorder of unregulated insulin secretion, leading to severe hypoglycemia in most cases. We previously described the adjunct use of alpelisib therapy in a 3-month-old patient with CHI. We now describe our observations in 2 additional patients with severe CHI treated with alpelisib therapy, resulting in discontinuation of all existing treatments and normalization of feeding. Two children (aged 3 and 4 years) with CHI (homozygous *ABCC8* and *KCNJ11* pathological variants) who were unresponsive to conventional therapies were treated with alpelisib. Treatment was initiated at 12.5 mg daily, with gradual dose adjustments based on clinical responses. Outcome measures included blood glucose variability, frequency of hypoglycemic episodes, need for supplemental feeding, and treatment safety. In both cases, alpelisib significantly improved glucose levels, reducing the frequency of hypoglycemic episodes. This allowed for the tapering and discontinuation of other medications (diazoxide and octreotide) and facilitated a transition to bolus gastrostomy-tube/oral feeding. No significant adverse effects were reported. Alpelisib shows promise as both an adjunctive and primary therapy for CHI, improving glucose levels and reducing dependence on continuous feeding and other medications. Randomized controlled trials are needed to assess its long-term safety and efficacy for CHI.

## Introduction

Congenital hyperinsulinism (CHI) is a rare and potentially life-threatening disorder characterized by excessive insulin secretion, resulting in persistent and severe hypoglycemia [1]. It is a leading cause of recurrent hypoglycemia in neonates and infants, with significant potential for neurological sequelae, including developmental delay, seizures, and even death if not promptly and adequately managed [2, 3].

Pathogenic variants in the *ABCC8* and *KCNJ11* genes (which encode the SUR1 and the KIR subunit of K_ATP_ channels, respectively) are the commonest genetic causes of CHI (variants in *ABCC8* are the most common). These pathogenic variants impair K_ATP_ channel function, leading to persistent depolarization of pancreatic β-cells and excessive insulin release. Patients with *ABCC8* or *KCNJ11* pathogenic variants present with severe, medically refractory CHI, necessitating aggressive interventions such as partial or near-total pancreatectomy [4, 5]. Usher-CHI syndrome is a distinct genetic condition linked to CHI. It arises from a homozygous deletion on chromosome 11p15, affecting both the *USH1C* and *ABCC8* genes, and these patients typically have severe CHI with sensorineural hearing loss and retinitis pigmentosa [6].

The first-line drug therapy for CHI is diazoxide; however, most patients with autosomal recessive pathological variants in *ABCC8 or KCNJ11* do not respond. Other treatment options include octreotide (both short and long acting), nifedipine, and sirolimus, but even with these therapies most patients will continue to have episodes of hypoglycemia [7, 8]. Alpelisib, a selective phosphatidylinositol 3-kinase (PI3K) inhibitor, plays a crucial role in regulating cell growth, insulin signaling, and glucose metabolism. Originally approved for treating *PIK3CA*-mutated, hormone receptor-positive advanced breast cancer [9] and overgrowth syndromes [10], alpelisib has also shown efficacy in managing non-islet cell tumor hypoglycemia [11]. A major side effect of alpelisib therapy in patients with *PIK3CA*-mutated, hormone receptor-positive advanced breast cancer is hyperglycemia (rarely diabetic ketoacidosis) [12]. We recently reported the adjuvant use of alpelisib in a 3-month-old patient with CHI who was unresponsive to conventional medical therapies [13]. After initiating alpelisib, the patient experienced a significant improvement in glucose levels. There was a reduction in the time spent below the glucose target threshold, and this allowed the discontinuation of continuous dextrose infusion, thus avoiding a near-total pancreatectomy. We now report on 2 additional patients treated with alpelisib whose blood glucose levels improved significantly; all other therapies were discontinued, allowing the patients to resume a normal feeding regimen.

## Case Presentation

### Case 1

This is a male child born at 36 weeks' gestation with a birth weight of 3.5 kg. There was no gestational diabetes mellitus in the mother and no other relevant family history. He presented postnatally with respiratory distress and severe hypoglycemic seizures, necessitating intubation and neonatal intensive care. He required a glucose infusion rate of up to 20 mg/kg/min to maintain blood glucose levels above 3.0 mmol/L (54 mg/dL).

### Case 2

This is a female patient born at term with a birth weight of 4.03 kg. Her mother was well in the pregnancy with no gestational diabetes mellitus. Immediately after birth, she presented with poor feeding, lethargy, and seizures secondary to severe hypoglycemia. The patient required a glucose infusion rate of 18 mg/kg/min to maintain glucose levels above 3.0 mmol/L (54 mg/dL). The patient had a significant family history of similar conditions, as her older sister, diagnosed with CHI, underwent pancreatectomy at 14 days of age.

## Diagnostic Assessment

### Case 1

The investigations in the neonatal period for hypoglycemia confirmed CHI (see Table 1), and the patient was started initially on diazoxide (maximum 15 mg/kg/day), octreotide (35 mcg/kg/day), and continuous high-calorie feeds via gastrostomy. At 6 months of age, he was switched to long-acting octreotide (35 mg given 4 times weekly). He then presented to our hospital at the age of 3 years. Biochemical investigations for the hypoglycemia reconfirmed CHI with a blood glucose level of 2.6 mmol/L (47 mg/d), insulin 127 pmol/L (18.2 µU/mL), and ketones 0.1 mmol/L. Genetic analysis identified a homozygous deletion in *USH1C* and *ABCC8* on chromosome 11p15.1 (Table 1). The genetic finding of a homozygous deletion in the *USH1C* and *ABCC8* genes suggested that this was diffuse CHI and that a focal lesion was unlikely, so no 18F-fluorodihydroxyphenylalanine positron emission tomography-computed tomography imaging was undertaken in his case. On assessment, he was on diazoxide (12 mg/kg/day), long-acting octreotide (35 mg given 4 times weekly), and 2 hourly bolus feeds via a gastrostomy tube. Any attempt to decrease the frequency of the bolus feeds led to the recurrence of hypoglycemia (as low as 2.9 mmol/L, 53 mg/dL) at least twice daily.

**Table 1.** Clinical, biochemical (at time of hypoglycemia in the neonatal period), and genetic findings in the 2 cases

Parameter	Case 1	Case 2	Normal range
Gestational age (weeks)	36 weeks	37 weeks	—
Birth weight (kg)	3.5 kg	4.03 kg	—
Insulin	45 pmol/L (6.3 µU/mL)	205 pmol/L (29.5 µU/mL)	18-173 pmol/L (2.6-25 µU/mL)
C-peptide	711 pmol/L (2.14 ng/mL)	953 pmol/L (2.86 ng/mL)	298-1324 pmol/L (0.9-4.0 ng/mL)
Glucose	1.7 mmol/L (30.6 mg/dL)	2.4 mmol/L (43.2 mg/dL)	3.9-5.6 mmol/L (70-100 mg/dL)
Ketone	0.2 mmol/L	0.1 mmol/L	<0.6 mmol/L
Cortisol	532 nmol/L (19.3 µg/dL)	490 nmol/L (17.8 µg/dL)	138-690 nmol/L (5-25 µg/dL)
GH	25 µg/L (25 ng/mL)	18 µg/L (18 ng/mL)	0-10 µg/L (0-10 ng/mL)
GIR (mg/kg/min)	20 mg/kg/min	18 mg/kg/min	4-8 mg/kg/min
Genetic finding	Arr [GRCh37] 11p15.1(17435236-17546308) (causes deletion of *USHIC* and *ABCC8*)	c.101G > A, p.Arg34His, Homozygous *KCNJ11*	

Abbreviation: GIR, glucose infusion rate.

### Case 2

The hypoglycemia screen in the neonatal period showed an inappropriate insulin level at the time of hypoglycemia, thus confirming CHI, and genetic analysis showed a homozygous *KCNJ11* pathogenic variant (Table 1). The patient was commenced on treatment with diazoxide. She did have an 18F-fluorodihydroxyphenylalanine positron emission tomography-computed tomography scan, which showed diffuse disease. Despite treatment with diazoxide and octreotide, her hypoglycemia persisted, leading to a near-total pancreatectomy at 22 days of age. Despite the near-total pancreatectomy, the patient continued to experience hypoglycemic episodes [at least 3 times a day to as low as 2.3 mmol/L (42 mg/dL)]. Her hypoglycemia was managed with continuous gastrostomy feeding during the night and 2 hourly bolus feeds during the day with long-acting octreotide injections at a dose of 30 mg every 4 weeks. Even on this regimen, she was still having episodes of daily hypoglycemia (2.5 mmol/L, 45 mg/dL). The patient also developed pancreatic exocrine insufficiency requiring pancreatin supplements and had marked food aversion with minimal oral intake.

This second patient presented at our hospital at the age of 4 years. Once again we reconfirmed the diagnosis of CHI with a blood glucose level of 2.1 mmol/L (38 mg/dL), insulin of 280 pmol/L (40 µU/mL, and ketones 0.1 mmol/L.

## Treatment

### Case 1

**Figure 1.**
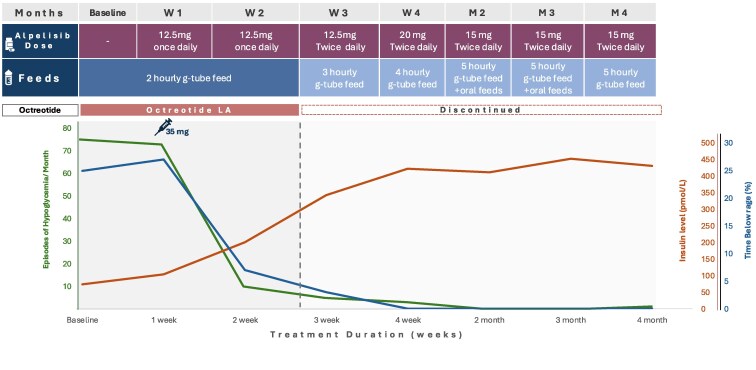
Treatment progress with alpelisib therapy over a 4-month period in case 1. The patient's treatment progress with alpelisib therapy, as reflected by changes in the serum insulin level and the percentage of time with glucose values below the target range of 63 mg/dL (3.5 mmol/L). To convert values for insulin to µU/mL, divide by 7.175. At 3 weeks, the patient was able to receive bolus G-tube feeding every 3 hours. Over time, the bolus G-tube feeding intervals were gradually spaced until reaching every 5 hours by the second month of treatment. The alpelisib dose was increased gradually until it reached 20 mg twice daily but was later reduced to 15 mg twice daily due to hyperglycemia. The percentage of time below the target glucose range dropped to 0% by 4 to 5 weeks. Continuous glucose monitoring was maintained throughout the observation period. Injections with octreotide long-acting release were discontinued in the second month of treatment.

Abbreviation: G-tube, gastrostomy tube.

Following approval from the Pharmaceutical and Therapeutics Committee of the hospital, we repurposed alpelisib therapy for this patient. Beginning with a low dose of 12.5 mg once daily, the dose was gradually increased to 20 mg twice daily. We observed a marked improvement in the glucose variability (time in range and frequency of hypoglycemia) by the fifth week (Fig. 1).

### Case 2

**Figure 2.**
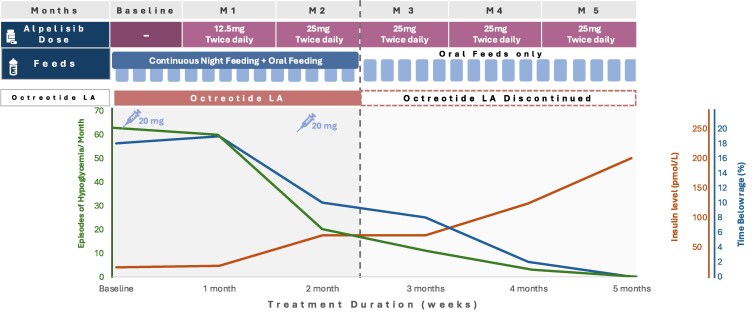
Treatment progress with alpelisib therapy over a 5-month period in case 2. The patient's treatment progress with alpelisib therapy, as reflected by changes in the serum insulin level and the percentage of time with glucose values below the target range of 63 mg/dL (3.5 mmol/L). To convert values for insulin to µU/mL, divide by 7.175. By 6 weeks the patient was able to discontinue continuous overnight feeding. The alpelisib dose was gradually increased until reaching 25 mg twice daily. Over time, the percentage of time below the target glucose range dropped to nearly 0% by month 5. Continuous glucose monitoring was maintained throughout the observation period. Injections with octreotide long-acting release were discontinued in the second month of treatment.

We commenced alpelisib therapy at 12.5 mg twice daily, and this was gradually increased to 25 mg twice daily. At 6 weeks we were able to stop her long-acting octreotide as well as her continuous overnight feeds (Fig. 2). Currently she is now feeding on demand, and her time in range is >99%. No side effects were observed in either patient while on the alpelisib therapy.

## Outcome and Follow up

The first patient’s weight and height remained along the 50th centile, while the second patient’s weight and height were along the 25th centile during follow-up. In both patients, there was no acanthosis nigricans or other clinical signs of insulin excess. No side effects were observed in either patient during alpelisib therapy except mild hyperglycemia in the first patient.

## Discussion

We first reported the adjunct use of alpelisib therapy in a 3-month CHI patient, avoiding the need for a near-total pancreatectomy [13]. Our observations in these 2 new patients suggest that alpelisib therapy also improves glucose levels significantly, allowing the discontinuation of other conventional medical therapies (such as diazoxide and octreotide), and may be used as the sole therapy. More importantly, in both patients the overnight gastrostomy feeds were stopped, and both children’s feeding patterns normalized. The first patient responded so well to alpelisib treatment that the dose had to be reduced (from 20 mg twice daily to 15 mg twice daily) due to occasional hyperglycemic episodes (>10 mmol/L, 180 mg/dL).

Alpelisib, a selective PI3K inhibitor, plays a crucial role in regulating cell growth, insulin signaling, and glucose metabolism. It has been approved for treating *PIK3CA*-mutated, hormone receptor-positive advanced breast cancer [9] and overgrowth syndromes in children over the age of 2 years [10]. Alpelisib has also shown efficacy in managing non-islet cell tumor hypoglycemia [11]. A major side effect of alpelisib therapy is hyperglycemia (in some cases diabetic ketoacidosis) and in some cases rashes and diarrhea [12].

Alpelisib is a small-molecule that, when administered orally, is rapidly absorbed [14]. Alpelisib is metabolized through various pathways, including hydrolysis, oxidation by CYP3A4, and glucuronidation by UGT1A9 [14]. The primary metabolite, M4, is inactive and nongenotoxic. Elimination is mainly through feces and, to a lesser extent, the urine, with over 90% excreted within 9 days. There are no pharmacological studies reported in children. The tissue specificity of alpelisib is influenced by the expression patterns of PI3Kα and the presence of *PIK3CA* mutations in different tissues [15]. While it effectively inhibits tumor growth in tissues with these mutations, its impact on normal tissues expressing PI3Kα can lead to some of the other side effects (diarrhea, rashes, and mouth ulcers). Therefore, understanding the distribution and activity of PI3Kα across various tissues is crucial for predicting both the therapeutic efficacy and potential toxicities of alpelisib [15].

The mechanism by which alpelisib improves blood glucose levels in CHI patients likely relates to its inhibition of the PI3K-AKT pathway, which is a key pathway in the insulin signaling cascade. Inhibition of PI3K signaling can reduce insulin signaling (despite increases in the serum insulin concentration), thereby improving blood glucose levels and mitigating hypoglycemia [9]. This effect has been demonstrated in other conditions, such as *PIK3CA*-related overgrowth syndromes and non-islet cell tumor hypoglycemia, where alpelisib has also been shown to improve glucose homeostasis [10, 11]. In our cases, this resulted in a marked reduction in the frequency of hypoglycemic episodes and a reduction in the need for continuous glucose supplementation and pharmacological interventions.

The safety profile of alpelisib in both cases was favorable, with no significant adverse effects observed during the treatment period. While some dose-dependent hyperglycemia was noted in the first patient, it was manageable by adjusting feeding schedules and alpelisib doses. We measured serum insulin levels weekly, and as expected, these were increased but did not increase the frequency of hypoglycemia. Given alpelisib's role in overgrowth syndromes, we conducted growth monitoring, including height and weight assessments. Both patients remained on their growth trajectories, with no evidence of growth attenuation or deceleration over the follow-up period. Furthermore, we assessed for insulin resistance-related sequelae, including acanthosis nigricans, hirsutism, and other signs of hyperinsulinemia (eg, rapid weight gain, metabolic disturbances), which until now were not observed in either patient, although long-term monitoring is necessary. These observations suggest that, with appropriate monitoring, alpelisib is a safe and effective treatment option for hyperinsulinism-associated hypoglycemia in pediatric patients.

In summary, our observations suggest alpelisib therapy improves blood glucose levels in CHI patients, allowing the discontinuation of other conventional treatments and normalization of feeding. Future randomized controlled trials should compare alpelisib with conventional therapies to establish its efficacy and safety across genetic subtypes and age groups. Additionally, studies on long-term outcomes, including potential effects on growth and neurodevelopment, are critical for integrating alpelisib into clinical practice.

## Learning Points

- Alpelisib can significantly improve glycemic control in patients with severe CHI.
- Alpelisib therapy may reduce or eliminate the need for other medications, such as diazoxide and octreotide.
- Alpelisib is a safe and effective treatment option for hyperinsulinism-associated hypoglycemia in pediatric patients.
- Future randomized controlled trials should compare alpelisib with conventional therapies to establish its efficacy and safety across genetic subtypes and age groups.

## Data Availability

Original data generated and analyzed for this case report are included in this published article.
